# Corneal Confocal Microscopy Features and Tear Molecular Profile in Study Participants with Discordance between Ocular Surface Disease Clinical Signs and Discomfort

**DOI:** 10.3390/jcm11092407

**Published:** 2022-04-25

**Authors:** Sharon D’Souza, Rohit Shetty, Archana Padmanabhan Nair, Ruchika Agrawal, Mor M. Dickman, Pooja Khamar, Rudy M. M. A. Nuijts, Arkasubhra Ghosh, Swaminathan Sethu

**Affiliations:** 1 Department of Cornea and Refractive Surgery, Narayana Nethralaya, Bangalore 560010, India; drsharondsouza@gmail.com (S.D.); drrohitshetty@yahoo.com (R.S.); dr.poojakhamar@gmail.com (P.K.); 2 GROW Research Laboratory, Narayana Nethralaya Foundation, Bangalore 560099, India; archana.nair@narayananethralaya.com (A.P.N.); ruchika.agrawal@narayananethralaya.com (R.A.); 3 Manipal Academy of Higher Education, Manipal 576104, India; 4 University Eye Clinic Maastricht, Maastricht University Medical Center, 6200 MD Maastricht, The Netherlands; mor.dickman@mumc.nl (M.M.D.); rudy.nuijts@mumc.nl (R.M.M.A.N.); 5 MERLN Institute for Technology-Inspired Regenerative Medicine, Maastricht University, 6200 MD Maastricht, The Netherlands

**Keywords:** ocular surface pain, in vivo confocal microscopy, corneal dendritic cells, microneuromas, dry eye disease, tear film

## Abstract

Various ocular surface conditions such as dry eye disease can present with severe discomfort and pain. However, it is clinically challenging to establish etiology and prescribe correct treatment in patients who have a lot of discordance between symptoms and signs. To understand the basis of such discordance, we stratified subjects with ocular surface pain based on concordance between the severity of signs and symptoms and evaluated corneal structural features and tear molecular factors. All subjects underwent slit lamp examination, dry eye evaluation, and ocular surface disease index (OSDI) scoring. Subjects were stratified into group 1—without symptoms or clinical signs; group 2—without symptoms but with signs; group 3—with similar severity of symptoms and signs; and group 4—with symptom severity greater than that of the signs. Laser scanning in vivo confocal microscopy (IVCM) and tear fluid analysis for soluble factors by multiplex ELISA was performed for all subjects. Patients with a higher grade of symptoms and signs showed increased corneal dendritic cell (cDC) density (*p* < 0.05) which was more pronounced in subjects with discordance between the symptoms and signs (group 4). A significantly higher proportion of microneuroma-like structures and cDC were observed in group 4. IL-17A levels were significantly elevated in the tears of subjects with more discomfort. Our results demonstrate that corneal IVCM and the measurement of tear film factors can help clinicians improve diagnosis and treatment choice. Stratifying patients with ocular surface discomfort on the basis of discordance between symptoms and clinical signs may help identify patients who need additional adjunctive targeted therapy to resolve their condition.

## 1. Introduction

Ocular pain and discomfort are common presenting symptoms for patients visiting the outpatient clinic and can significantly deteriorate a patient’s quality of life and their activities of daily living [1,2]. Infections, inflammation, and raised intraocular pressure are some common aetiologies of ocular pain in general, while conditions such as dry eye disease (DED), corneal infections, or injury are often related to ocular surface pain [3]. Structural factors such as the dense innervation of the cornea as well as the immune, inflammatory, and neural interconnections of the ocular surface have an important role to play in ocular surface pain [3,4]. In some cases, the pain may be disproportionately higher than the clinical signs, thereby complicating the diagnosis and making management challenging [5]. Exaggerated ocular surface pain can be secondary to inflammation, increased nociception, neuropathy, or a combination of these factors that may co-exist with various ocular surface conditions. Nociceptive pain usually occurs due to an alteration in a non-neural tissue resulting in an increase in nociceptive molecular factors [1], whereas neuropathic corneal pain (NCP) is secondary to the involvement of the somatosensory nervous system [6]. However, significant overlap between these entities can occur [7]. It is important to identify nociceptive and neuropathic aspects of pain so as to treat them appropriately [8]. Neuropathic cases can have symptoms of pain and discomfort despite a complete absence of clinically visible ocular surface abnormalities [9,10]. Overlap between conditions and this discordance between signs and symptoms are major challenges in treating these patients even in those with an established diagnosis [11,12,13]. In addition, many patients have an assumed diagnosis of DED, and do not respond to therapy as expected. These patients are often treated with multiple different topical and systemic medications with suboptimal relief and may also get referred to the pain management clinic or for a psychological assessment [14].

A possible reason for this exaggerated pain response without significant clinical features may be subclinical inflammation [15,16], which may involve the corneal nerves leading to complaints of pain, discomfort, and a burning sensation secondary to polymodal nociceptor and thermoreceptor activation [17,18]. Additional tests such as laser scanning in vivo confocal microscopy (IVCM) and tear molecular analysis can help identify these hidden causes for pain. IVCM is an excellent tool to assess both inflammation and nerve abnormalities by assessing the density and morphology of corneal dendritic cells (cDC) and the alterations in the corneal nerve structures that are known to be associated with ocular surface pain [19,20]. Chronic dry eye and ocular surface inflammation can also have recalcitrant symptomatology even with an established diagnosis due to changes in corneal nerves structure and function resulting in NCP [21]. Therefore, evaluating patients with poor correlation between symptoms and signs would aid in stratifying treatment. As disparity between symptoms and signs is an important feature of NCP, we have used this as the main criteria to divide patients into four groups. We therefore evaluated the corneal IVCM feature changes (cDC, sub-basal nerve plexus features—SBNP, and microneuroma-like structures) and tear molecular factors. We have then analysed the variations between these groups to identify specific subgroup characteristics that would help treatment.

## 2. Materials and Methods

### 2.1. Study Cohort, Clinical Examination, and Study Groups

This cross-sectional study has received approval of the Narayana Nethralaya Institutional ethics board. Subject recruitment, clinical information, and sample collection procedures were conducted in accordance with the tenets of the Declaration of Helsinki. Written informed consent was obtained from each subject prior to inclusion in the study. A total of 145 subjects (287 eyes) were included in the study with 115 patients (229 eyes) having the primary symptom of ocular surface pain or discomfort referred to the Cornea Clinic of Narayana Nethralaya, located in Bangalore, India, and 30 control subjects (58 eyes) with no symptoms of ocular pain or discomfort and no clinically visible ocular abnormality on examination. Patients were evaluated for possible causes for ocular discomfort using a detailed clinical history, including history of systemic illness and ocular examination. Visual acuity assessment, refraction, detailed slit-lamp examination including ocular surface evaluation for signs of inflammation and eyelid and meibomian gland assessment, and fundus evaluation were performed to exclude other ocular comorbidities. Exclusion criteria included the use of contact lenses, ocular infection in the last six months, uveitis, ocular trauma, history of any ocular surgery including refractive surgery, cicatrizing disease, lid abnormalities and those on topical steroids and topical anti-inflammatory or immunomodulatory medication including cyclosporine A in the last 3 months.

As there are many possible causes for ocular surface pain, a detailed assessment is required to arrive at the possible underlying diagnosis and plan the appropriate management [22]. The presenting symptom of pain or discomfort was assessed using the ocular surface disease index (OSDI) questionnaire (Allergan, Dublin, Ireland) [23,24], and the severity graded based on the standard scores as follows: normal < 12; mild 13–22; moderate 23–32; and severe > 32 [25]. As DED is one the most common causes for ocular surface pain [8] and referral to the cornea clinic, detailed ocular surface and dry eye evaluation was done. Dry eye evaluation included Schirmer’s test without anaesthesia, tear film breakup time (TBUT), and corneal and conjunctival fluorescein staining. Using sterile Schirmer’s strips (Contacare Ophthalmics and Diagnostics, Vadodara, Gujarat, India), the Schirmer’s test was performed. TBUT and ocular surface staining were performed using fluorescein strips (Contacare Ophthalmics and Diagnostics). Clinical tests such as the ocular surface staining with fluorescein and other dyes give us an insight into the health of the ocular surface. Tear film break up time assesses the tear film stability and the Schirmer’s test measures the tear secretion rate. Subjects were divided into those without DED or having mild, moderate, and severe DED based on the Schirmer’s and/or TBUT values as per standard guidelines from the TFOS DEWSII (Tear Film & Ocular Surface Society Dry eye workshop II) [26,27]. Further, DED patients have been classified as per TFOS DEWII into two predominant categories, i.e., aqueous deficient dry eye disease (ADED) or evaporative dry eye disease (EDED) based on the Schirmer’s test I value and TBUT. Subjects with TBUT < 10 s but with a Schirmer’s test I value > 10 mm/5 min were grouped as EDED. Subjects with a Schirmer’s test I value < 10 mm/5 min were classified as ADED. Grade of tear film instability assessed by TBUT are as follows: normal ≥ 10 s, mild 7–9 s, moderate 5–7 s, and severe < 5 s. Grade of tear fluid secretion status based on Schirmer’s test I metrics are as follows: normal ≥ 10 mm/5 min, mild 7–9 mm/5 min, moderate 5–7 mm/5 min, and severe < 5 mm/5 min.

**Figure 1:**
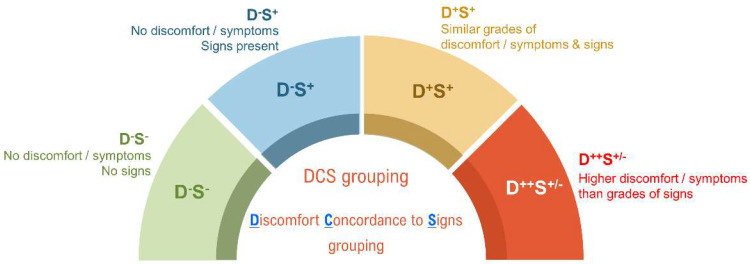
Discomfort concordance to signs (DCS) grouping. Schematic representation of the various groups under which the study subjects were categorized based on the concordance between the status of discomfort/symptoms (D) and signs (S). Group 1 (D^-^S^-^) include subjects without discomfort/symptoms (OSDI score: <12) and no clinical signs (TBUT: ≥10 s, Schirmer’s test: ≥10 mm/5 min), hence, presumed as normal or control subjects. Group 2 (D^-^S^+^) include subjects without discomfort/symptoms (OSDI score: <12) but present with signs (TBUT: <10 s and/or Schirmer’s test: < 10 mm/5 min). Group 3 (D^+^S^+^) include subjects with similar grades of discomfort/symptoms and signs. Group 4 (D^++^S^+/-^) included subjects in whom the discomfort/symptom grade is higher than the grade/severity of signs. OSDI severity scale (Normal < 12; Mild, 13–23; Moderate, 24–32; Severe > 32). TBUT severity scale (Normal ≥ 10 s; Mild 7–9 s; Moderate 5–7 s; Severe < 5 s). Schirmer’s test severity scale (Normal ≥ 10 mm/5 min; Mild 7–9 mm/5 min; Moderate 5–7 mm/5 min; Severe < 5 mm/5 min).

To determine additional factors that may contribute to the ocular surface pain, the study participants were grouped based on the disparity between the severity of their symptoms and signs irrespective of their dry eye status, as described in discomfort concordance to signs (DCS) grouping (Figure 1). Group 1 (D^-^S^-^)-presumed normal or control subjects no discomfort D^-^ (OSDI score: <12) and no clinical signs S^-^ (TBUT: ≥10 s, Schirmer’s test: ≥10 mm/5 min, no ocular surface abnormality). Group 2 (D^-^S^+^) includes subjects without discomfort D^-^ (OSDI score: normal < 12) but with signs S^+^ (TBUT: <10 s and/or Schirmer’s test: <10 mm/5 min and/or ocular surface staining). Group 3 (D^+^S^+^) includes subjects with similar grades of discomfort D^+^ and signs S^+^ as per the standard gradings discussed previously such as moderate symptoms on OSDI and moderate severity of signs on DED evaluation. Group 4 (D^++^S^+/-^) includes subjects with disparate symptoms and signs, having symptom grade very high D^++^ and signs low or absent S^+/-^. IVCM parameters (cDC density, sub-basal nerve plexus morphologic characteristics, and microneuroma-like structures) and tear fluid soluble factor levels were studied across all 4 groups.

### 2.2. In Vivo Confocal Microscopy (IVCM) Imaging

**Figure 2:**
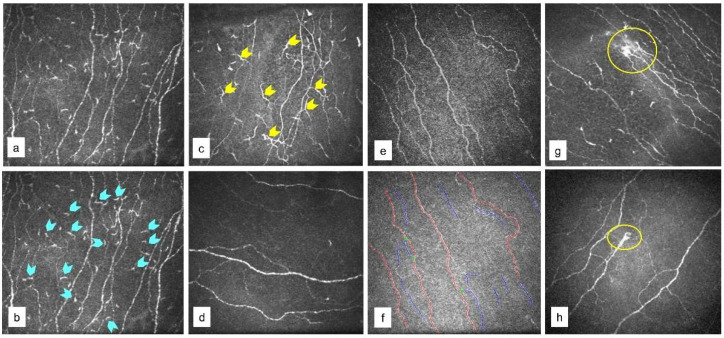
Corneal dendritic cells, microneuroma-like structures, and sub-basal nerve plexus in cornea of study participants. Panels are representative in vivo confocal microscope images showing (**a**–**c**) corneal dendritic cells, (**d**–**f**) corneal sub-basal nerve plexus and (**g**,**h**) microneuroma-like structure indicated within the yellow circle. Immature forms of dendritic cells are shown with blue arrows in (**b**). Mature forms of dendritic cells are indicated with yellow arrows in (**c**). The blue and red lines shown in (**f**) indicates CC metrics software detection of the nerves to determine the various morphological parameters.

IVCM is a non-invasive imaging modality that can study the corneal microscopic structure, nerves, and cDC density and morphology [28,29]. IVCM was performed using the Rostock Corneal Module/Heidelberg Retina Tomograph ll (RCM/HRT ll; Heidelberg Engineering GmBH, Dossenheim, Germany). Proparacaine drops (0.5%) were instilled prior to the procedure to anaesthetize the ocular surface. The captured ICVM images that passed image quality control were used for analysis. cDC were identified based on their morphology and categorized into mature and immature [30] as shown in Figure 2a–c. cDC density (cDCD) expressed in cells/mm^2^ was quantified using Cell Count software (Heidelberg Engineering GmbH) [31,32]. Sub-basal nerve plexus (SNBP) features (Figure 2d–f) in the study subjects were also determined as previously described [32]. Corneal nerve fibre area (CNFA) per square millimetre, corneal nerve fibre length (CNFL) in millimetres per square millimetre, corneal nerve fibre density (CNFD) per square millimetre, corneal nerve fibre width (CNFW) per square millimetre, corneal nerve branch density (CNBD) per square millimetre, and total branch density (CTBD) per square millimetre were analysed in IVCM images using Automatic CCMetrics software, version 1.0 (University of Manchester, Manchester, UK) [31,32]. Microneuroma-like structures in the nerve plexus were identified (Figure 2g,h) as described in existing literature [1,33,34,35].

### 2.3. Tear Fluid Collection

Tear fluid samples were collected from the study subjects using Schirmer’s strips as per standard protocol [36] and stored in 1.5 mL microcentrifuge tubes at −80 °C until further processing. On the day of analysis, the tear fluid was extracted from Schirmer’s strips by agitation in 300 µL sterile 1× PBS for 2 h at 4 °C. The tear fluid proteins were eluted by centrifugation and immediately used for the downstream experiment as mentioned in Section 2.4 to measure the various soluble factors by multiplex ELISA.

### 2.4. Soluble Factors Level Measurement

The concentration of secreted factors in the tear fluid that was measured include Interleukin (IL)-1α, IL-1 β, IL-2, IL-6, IL-8, IL-10, IL-17A, IFNγ (Interferon gamma), MCP1/CCL2 (Monocyte chemoattractant protein 1), RANTES/CCL5 (Regulated upon Activation, Normal T cell Expressed), sICAM1 (soluble Intercellular adhesion molecule 1), and TNFα (Tumor necrosis factor alpha and VEGF-A (Vascular endothelial growth factor—A). The levels of these secreted factors in the tears were measured by multiplex ELISA using cytometric bead array (BD Biosciences, San Jose, CA, USA) on a flow cytometer (BD FACSCantoII, BD Biosciences, San Jose, CA, USA) [36]. BD FACSDiva software (BD Biosciences, San Jose, CA, USA) was used to acquire the bead–antibody conjugate–analyte complexes and record their signal intensities. FCAP array version 3.0 (BD Biosciences, San Jose, CA, USA) was used to determine absolute concentration of the analytes using respective standards. The wetting length of the Schirmer’s strip noted during tear collection and tear protein elution buffer volume were used for calculation of the dilution factor to derive the normalized concentration values of tear analytes.

### 2.5. Statistical Analyses

The normality of data was assessed by the Shapiro–Wilk normality test. The Kruskal–Wallis test with Dunn’s multiple comparison test and the Mann–Whitney test were used to analyse the differences in the variable between the study groups in the datasets. Statistical analyses were performed with GraphPad Prism 6.0 (GraphPad Software, Inc., La Jolla, CA, USA). *p* < 0.05 was considered statistically significant.

## 3. Results

### 3.1. Ocular Surface Clinical Parameters, IVCM Features, and Tear Soluble Factors in Different Groups

**Figure 3:**
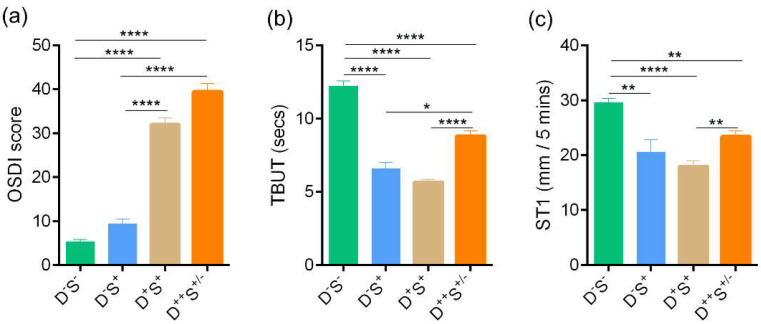
Ocular surface disease index score, Tear break up time, and Schirmer’s test values in subjects categorized based on discomfort concordance to signs (DCS) grouping strategy. Bar graphs represent (**a**) Ocular surface disease index (OSDI) scores indicative of the discomfort/symptoms, (**b**) Tear break up time (TBUT) values in secs—indicative of sign and (**c**) Schirmer’s test (ST1) values in mm/5 min—indicative of sign in the study subjects grouped in the various categories based on DCS grouping strategy. Group 1 (D^-^S^-^): n = 58 eyes; Group 2 (D^-^S^+^): n = 28 eyes; Group 3 (D^+^S^+^): n = 127 eyes; Group 4 (D^++^S^+/-^): n = 74 eyes. * *p* < 0.05, ** *p* < 0.01, **** *p* < 0.0001. Kruskal–Wallis test with Dunn’s multiple comparisons test was performed. Age (D^-^S^-^, median (range) 32.5 (25–73) yrs; D^-^S^+^, 31 (26–54) yrs; D^+^S^+^, 38 (22–72) yrs, and D^++^S^+/-^, 36 (20–65) yrs). Sex distribution (D^-^S^-^, M/F 16/14; D^-^S^+^, M/F 4/10; D^+^S^+^, M/F 32/32, and D^++^S^+/-^, M/F 18/9).

Patients were divided into the four groups based on the parameters discussed previously. Group 1 (D^-^S^-^): n = 58 eyes; Group 2 (D^-^S^+^): n = 28 eyes; Group 3 (D^+^S^+^): n = 127 eyes; and Group 4 (D^++^S^+/-^): n = 74 eyes. The age distribution of subjects in the various groups are as follows: median (range)—D^-^S^-^, 32.5 (25–73) years; D^-^S^+^, 31 (26–54) years; D^+^S^+^, 38 (22–72) years, and D^++^S^+/-^, 36 (20–65) years. The sex distribution of subjects in the various groups are as follows: D^-^S^-^—M/F 16/14; D^-^S^+^—M/F 4/10; D^+^S^+^—M/F 32/32; and D^++^S^+/-^—M/F 18/9. Patients in the D^+^S^+^ (group 3) and D^++^S^+/-^ (group 4) had a significantly higher symptom grade compared to both the D^-^S^-^ (group 1) and D^-^S^+^ (group 2) (Figure 3a). The tear break up time (TBUT) and the Schirmer’s test values were significantly lower in D^+^S^+^ and D^++^S^+/-^ compared to the normal D^-^S^-^ group (Figure 3b,c). The TBUT and Schirmer’s test values were significantly worse in the D^+^S^+^ even though the symptoms were worst in the D^++^S^+/-^ group (Figure 3b,c), which suggests that some patients in the highly symptomatic D^++^S^+/-^ group could have only mild or no DED. A total of 201 out of 229 eyes had varying grades of DED. Ocular surface staining was noted in 13.8% of all eyes included in the study (35/287 eyes) of which most had varying grades of DED. Additional signs of ocular surface inflammation such as congestion or staining were also noted.

**Figure 4:**
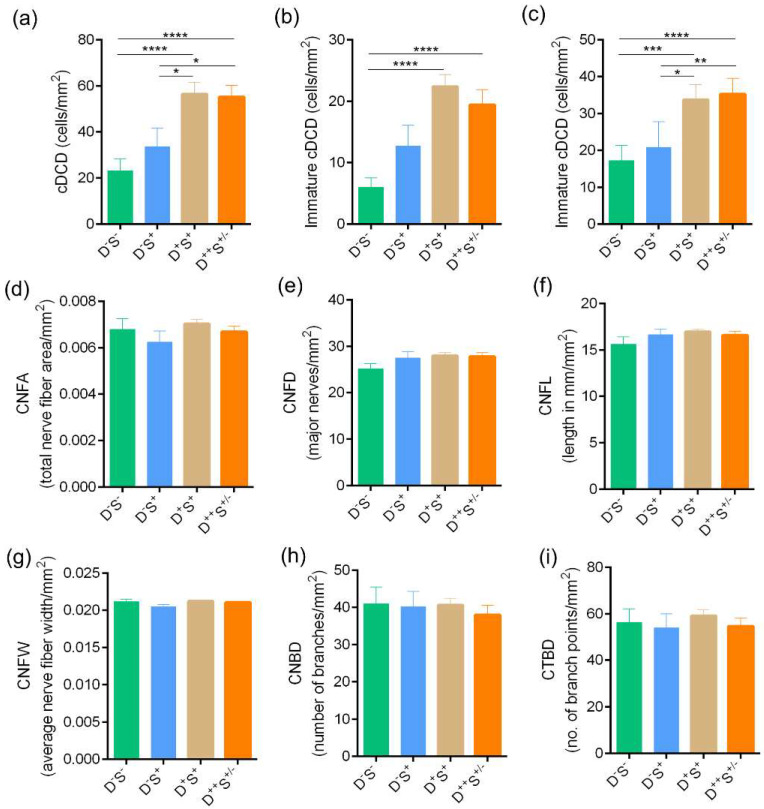
Corneal dendritic cell density and sub-basal nerve plexus features profile in subjects categorized based on discomfort concordance to signs (DCS) grouping strategy. Bar graphs represent (**a**) total corneal dendritic cell density (cDCD), (**b**) density of immature forms of corneal dendritic cells, (**c**) density of mature forms of corneal dendritic cells, (**d**) corneal nerve fibre area—CNFA, (**e**) corneal nerve fibre density—CNFD, (**f**) corneal nerve fibre length—CNFL, (**g**) corneal nerve fibre width—CNFW, (**h**) corneal nerve branch density—CNBD and (**i**) corneal total branch density—CTBD determined using laser scanning in vivo confocal microscopic images in the study subjects grouped in the various categories based on DCS grouping strategy. The number of eyes analysed for corneal dendritic cell density are as follows: Group 1 (D^-^S^-^): n = 58 eyes; Group 2 (D^-^S^+^): n = 28 eyes; Group 3 (D^+^S^+^): n = 127 eyes; Group 4 (D^++^S^+/-^): n = 74 eyes. The number of eyes analysed for sub-basal nerve plexus features are as follows: Group 1 (D^-^S^-^): n = 27 eyes; Group 2 (D^-^S^+^): n = 17 eyes; Group 3 (D^+^S^+^): n = 98 eyes; Group 4 (D^++^S^+/-^): n = 55 eyes. * *p* < 0.05, ** *p* < 0.01, *** *p* < 0.001, **** *p* < 0.0001, Kruskal–Wallis test with Dunn’s multiple comparisons test was performed.

The density of total, immature, and mature forms of cDC were significantly higher in the D^+^S^+^(group 3) and D^++^S^+/-^ (group 4) group compared to the D^-^S^-^ (normal) group 1 (Figure 4a–c). No significant difference in the cDC density was observed between the D^+^S^+^ group 3 and D^++^S^+/-^ group 4 (Figure 4a–c).

**Figure 5:**
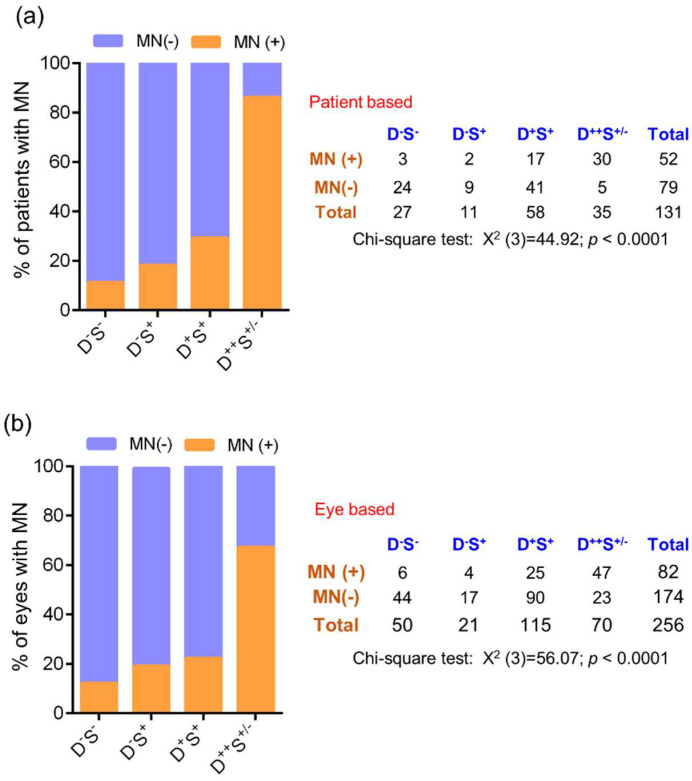
Frequency of microneuroma-like structures in subjects categorized based on discomfort concordance to signs (DCS) grouping strategy. Stacked bar graphs represent (**a**) percentage of patients with and without microneuroma-like structures determined in laser scanning in vivo confocal microscopy images in the different groups. The adjacent table provides the absolute number of patients with and without microneuroma-like structures in the different groups. (**b**) percentage of eyes with and without microneuroma-like structures determined in laser scanning in vivo confocal microscopy images in the different groups. The adjacent table provides the absolute number of eyes with and without microneuroma-like structures in the different groups. Chi-square test was performed to determine the statistical significance of the difference in the frequency of microneuroma-like structures between the groups. *p* < 0.05 is considered to be statistically significant. MN—microneuroma-like structures.

**Figure 6:**
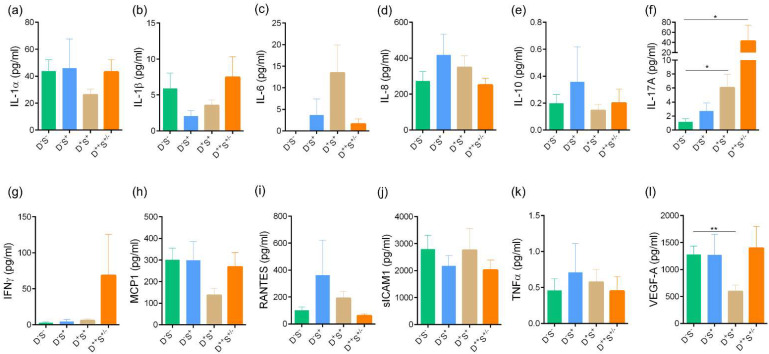
Tear fluid secreted factors level in subjects categorized based on discomfort concordance to signs (DCS) grouping strategy. Bar graphs represent the tear fluid levels of (**a**) IL-1α, (**b**) IL-1β, (**c**) IL-6, (**d**) IL-8, (**e**) IL-10, (**f**) IL-17A, (**g**) IFNγ, (**h**) MCP1, (**i**) RANTES, (**j**) sICAM1, (**k**) TNFα, and (**l**) VEGF-A in the study subjects grouped in the various categories based on DCS grouping strategy. IL—Interleukin, IFNγ—Interferon gamma, MCP1/CCL2—Monocyte chemoattractant protein 1, RANTES/CCL5—Regulated upon Activation, Normal T cell Expressed, and Secreted, sICAM1—soluble Intercellular adhesion molecule 1, TNFα—Tumor necrosis factor alpha, and VEGF-A—Vascular endothelial growth factor—A. pg/mL—picogram per millilitre. Group 1 (D^-^S^-^): n = 28 eyes; Group 2 (D^-^S^+^): n = 11 eyes; Group 3 (D^+^S^+^): n = 35 eyes; and Group 4 (D^++^S^+/-^): n = 16 eyes. * *p* < 0.05, ** *p* < 0.01, Kruskal–Wallis test with Dunn’s multiple comparisons test was performed.

A high proportion of microneuroma-like features were seen in group 3 D^+^S^+^ and group 4 D^++^S^+/-^ groups, with a significant increase in the proportion of microneuroma-like structures observed in the disparity group D^++^S^+/-^ group (Group 4) compared to other groups (Figure 5). No significant difference in the various corneal sub-basal nerve plexus morphological parameters was observed between the groups (Figure 4d–i). Among the various tear fluid soluble factors (cytokines, chemokines, soluble cell adhesion molecules, and growth factors) measures, the level of IL-17A was observed to be significantly higher in subjects in group 3 D^+^S^+^ and group 4 D^++^S^+/-^ group compared to the normal D^-^S^-^ group 1 (Figure 6f). A higher level of IL-17A was observed in the D^++^S^+/-^ group 4 compared to the D^+^S^+^ group 3 (Figure 6f). A significantly lower level of VEGF-A was observed in the D^+^S^+^ group 3 compared to the D^-^S^-^ group 1 (Figure 6l).

### 3.2. Ocular Surface Clinical Parameters, IVCM Features, and Tear Soluble Factors in Subjects with High Ocular Surface Discomfort but no Clinical Signs (D^++^S^-^)

**Figure 7:**
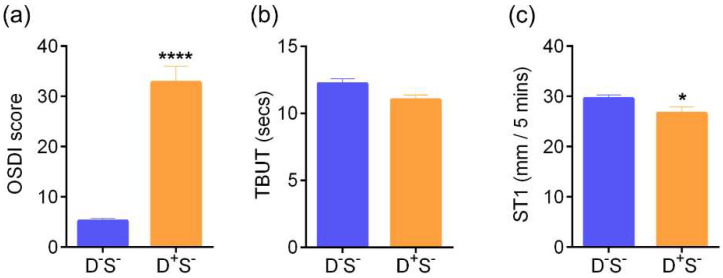
Ocular surface disease index score, Tear break up time, and Schirmer’s test values in subjects with discomfort but no signs. Bar graphs represent (**a**) Ocular surface disease index (OSDI) scores indicative of the discomfort/symptoms, (**b**) Tear break up time (TBUT) values in secs—indicative of sign and (**c**) Schirmer’s test (ST1) values in mm/5 min—indicative of sign in the study subjects. D^-^S^-^ indicates subjects without discomfort/symptoms and signs (n = 58 eyes) and D^+^S^-^ indicates subjects with discomfort/symptoms but no signs (n = 28 eyes). * *p* < 0.05, **** *p* < 0.0001, Mann–Whitney Test was performed. Age (D^-^S^-^, median (range) 32.5 (25–73) yrs and D^+^S^-^, 30 (20–57) yrs). Sex distribution (D^-^S^-^, M/F 16/14 and D^+^S^-^, M/F 4/10).

**Figure 8:**
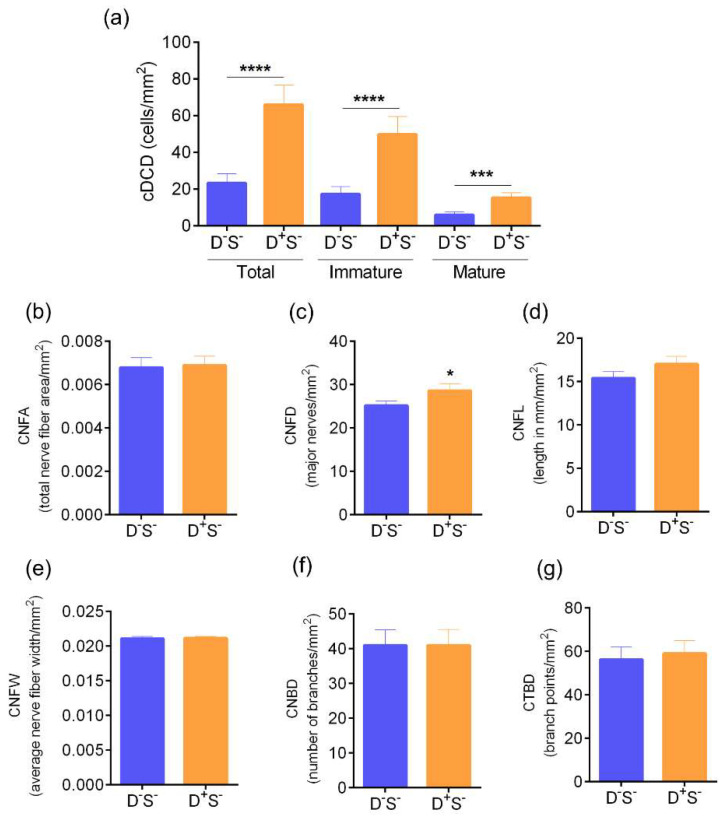
Corneal dendritic cell density and sub-basal nerve plexus feature profile in subjects with discomfort but no signs. Bar graphs represent (**a**) profile of total, immature and mature forms of corneal dendritic cell density (cDCD), (**b**) corneal nerve fibre area—CNFA, (**c**) corneal nerve fibre density—CNFD, (**d**) corneal nerve fibre length—CNFL, (**e**) corneal nerve fibre width—CNFW, (**f**) corneal nerve branch density—CNBD, and (**g**) corneal total branch density—CTBD determined in laser scanning in vivo confocal microscopy images in the study subjects. D^-^S^-^ indicates subjects without discomfort/symptoms and signs and D^+^S^-^ indicates subjects with discomfort/symptoms but no signs. (**a**): D^+^S^-^, n = 58 eyes and D^+^S^-^, n = 28 eyes. *** *p* < 0.001, **** *p* < 0.0001, Mann–Whitney Test. (**b**–**g**): D^+^S^-^, n = 29 eyes and D^+^S^-^, n = 19 eyes. * *p* < 0.05, Mann–Whitney Test was performed.

**Figure 9:**
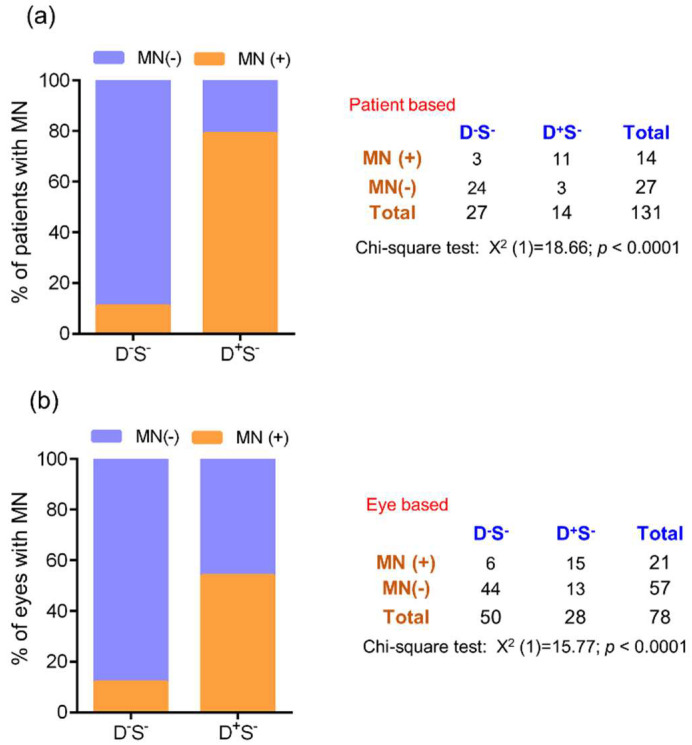
Frequency of microneuroma-like structures in subjects with discomfort but no signs. Stacked bar graphs represent (**a**) percentage of patients with and without microneuroma-like structures determined in laser scanning in vivo confocal microscopy images in the different groups. The adjacent table provides the absolute number of patients with and without microneuroma-like structures in the different groups. (**b**) percentage of eyes with and without microneuroma-like structures determined in laser scanning in vivo confocal microscopy images in the different groups. The adjacent table provides the absolute number of eyes with and without microneuroma-like structures in the different groups. D^-^S^-^ indicates subjects without discomfort/symptoms and signs and D^+^S^-^ indicates subjects with discomfort/symptoms but no signs. Chi-square test was performed to determine the statistical significance of the difference in the frequency of microneuroma-like structures between the groups. *p* < 0.05 is considered to be statistically significant. MN—microneuroma-like structures.

The OSDI scores were significantly higher in D^++^S^-^ compared to D^-^S^-^ (control) groups. (Figure 7a). The TBUT and Schirmer’s test values were within the normal range (Figure 7b,c). The density of total, immature, and mature forms of cDC were significantly higher in D^++^S^-^ compared to the control D^-^S^-^ group (Figure 8a). The corneal nerve fibre density was observed to be significantly higher in D^++^S^-^ compared to D^-^S^-^ (Figure 8c). No other significant difference in the various corneal sub-basal nerve plexus morphological parameters was observed between the groups (Figure 8b,d–g). The proportion of subjects and eyes with microneuroma-like structures were significantly higher in the D^++^S^-^ group 4 compared to the D^-^S^-^ (Figure 9). It is to be noted that the VEGF-A level was markedly lower (*p* = 0.07) in D^++^S^-^ compared to D^-^S^-^ groups (Table 1). However, no significant differences in the tear soluble factors were observed between D^++^S^-^ compared to D^-^S^-^ groups (Table 1).

### 3.3. Ocular Surface Clinical Parameters, IVCM Features and Tear Soluble Factors in Subjects with DED (Evaporative or Aqueous Deficient)

**Figure 10:**
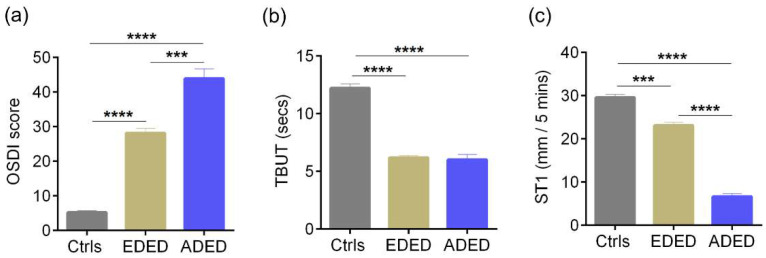
Ocular surface disease index score, Tear break up time, and Schirmer’s test values in subjects with dry eye disease. Bar graphs represent (**a**) Ocular surface disease index (OSDI) scores indicative of the discomfort/symptoms, (**b**) Tear break up time (TBUT) values in secs—indicative of sign and (**c**) Schirmer’s test (ST1) values in mm/5 min—indicative of sign in controls and in subjects with evaporative dry eye disease (EDED) or aqueous deficient dry eye disease (ADED). Controls are subjects without discomfort/symptoms and signs (D^-^S^-^). Controls (Ctrls): n = 58 eyes; EDED: n = 147 eyes; ADED: n = 48 eyes. *** *p* < 0.001, **** *p* < 0.0001, Kruskal–Wallis test with Dunn’s multiple comparisons test was performed. Age (controls, median (range) 32.5 (25–73) yrs, EDED, 35.5 (20–65) yrs, and ADED, 46 (23–72) yrs). Sex distribution (controls, M/F 16/14, EDED, M/F, 35/39, and ADED, M/F 13/11).

**Figure 11:**
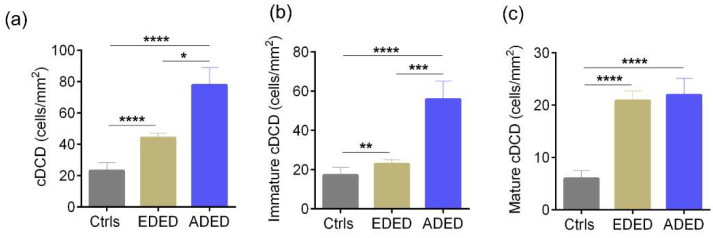
Corneal dendritic cell density in subjects with dry eye disease. Bar graphs represent (**a**) total corneal dendritic cell density (cDCD), (**b**) density of immature forms of corneal dendritic cells and (**c**) density of mature forms of corneal dendritic cells, determined using laser scanning in vivo confocal microscopic images in controls and in subjects with evaporative dry eye disease (EDED) or aqueous deficient dry eye disease (ADED). Controls are subjects without discomfort/symptoms and signs (D^-^S^-^). The number of eyes analysed for corneal dendritic cell density are as follows: Controls (Ctrls): n = 52 eyes; EDED: n = 144 eyes; ADED: n = 48 eyes. The number of eyes analysed for sub-basal nerve plexus features are as follows: Controls (Ctrls): n = 29 eyes; EDED: n = 114 eyes; ADED: n = 32 eyes. * *p* < 0.05, ** *p* < 0.01, *** *p* < 0.01, **** *p* < 0.0001, Kruskal–Wallis test with Dunn’s multiple comparisons test was performed.

The age distribution of subjects in the various groups are as follows: median (range)—controls 32.5 (25–73) years, evaporative dry eye disease—EDED 35.5 (20–65) years, and aqueous deficient dry eye disease—ADED 46 (23–72) years. The sex distribution of subjects in the various groups are as follows: controls—M/F 16/14, EDED—M/F, 35/39, and ADED—M/F 13/11. The OSDI scores were significantly higher in both EDED and ADED subjects compared to the controls, and the OSDI score in ADED was significantly higher compared to EDED as well (Figure 10a). The TBUT and Schirmer’s test values were significantly lower in EDED and ADED subjects compared to the controls (Figure 10b,c). The density of total, immature, and mature forms of cDC were significantly higher in both EDED and ADED compared to the controls (Figure 11a–c). The density of the total and immature forms of cDC were significantly higher in ADED compared to EDED (Figure 11a,b). No significant difference in the sub-basal nerve plexus parameters in DED compared to the controls was found (Supplementary Figure S1). However, the proportion of subjects and eyes with microneuroma-like structures in corneal nerves were significantly higher in DED compared to the controls (Supplementary Figure S2). The levels of IL-6, IL-17A, RANTES, and MCP1 were significantly higher and VEGF-A significantly lower in EDED and/or ADED compared to the controls (Table 2).

## 4. Discussion

Our understanding of acute and chronic ocular surface discomfort and pain continues to evolve. In patients who have a clinically attributable and comparable symptoms, the treatment is more straight forward and directed at the underlying aetiology. However, at the cornea clinic, we observe a large number of patients who have a disparity between clinical signs and symptoms where the severity of the symptoms cannot be explained by clinically visible signs. This can be due to an underlying nociceptive and neuropathic pain component [35]. Neuropathic corneal pain may not have clinically evident signs and has been classically referred to as “pain without stain” [12]. Thus, the pathophysiology of such exaggerated discomfort could be related to ocular surface inflammation, altered nociception, or neuropathy [1,37]. The perception of pain on the ocular surface is likely an interplay between the structural, epithelial, neuronal, molecular, and immune cell changes in the eye and neuronal connections to higher centres in the thalamus and somatosensory cortex [38,39]. Structurally, as the cornea has the highest nerve density in the body, it is particularly sensitive to alterations in the local molecular factors and environmental influences [40]. The status of features such as the cDC density, the sub-basal nerve plexus, microneuroma-like structures, and tear fluid factors needs to be characterized comprehensively in subjects with discordant signs and symptoms. The current study addresses this knowledge gap by objectively stratifying patients based on the discordance between the severity of ocular surface signs and symptoms and determining how cornea-specific features vary within groups.

DED status, and ocular surface clinical status and discomfort (OSDI) scores for each patient were evaluated. The chronicity of the pain and the patient’s description of the discomfort or pain were also taken into consideration [14]. When evaluating the severity of symptoms across the groups we found that the OSDI score was significantly higher in D^+^S^+^ (group 3) and D^++^S^+/-^ (group 4) as compared to D^-^S^-^ (group 1, normal) subjects. The cause for the increased pain in this group of patients could be due to the inflammation, altered nociception, or neuropathy. Chronic DED and the associated inflammation are common causes for increased nociception or NCP. The clinical signs were most significantly altered in the D^+^S^+^ group 3 (where the severity of symptoms and signs are proportionate). In the D^++^S^+/-^ group 4, where symptoms are out of proportion to signs, the alteration to DED clinical parameters were relatively less severe. These findings also corroborate those seen in other studies that state that increased nociception and NCP can be associated with different ocular and systemic conditions [41,42]. This reiterates the hypothesis that there may be additional factors driving the discomfort in this discordant group, hence we analysed IVCM and tear molecular factors across the cohort.

We found a significant increase in cDC density in D^+^S^+^ group 3 and D^++^S^+/-^ group 4 compared to normal D^-^S^-^. The highest cDC density was seen in the D^++^S^+/-^ group, thereby implying that the cDC density correlates positively with the patient’s discomfort. This finding is supported by previous studies that reported an association of cDC density with increased inflammation and discomfort [36,43]. The cDC may proliferate within a tissue and secrete a variety of inflammatory factors that can raise the overall nociceptive response [44]. Corneal sub-basal nerve fibre features have been studied in ocular surface conditions including DED with varied observations [45]. A significant increase in cDC density has been reported in a metanalysis on IVCM features similar to the observations made in the current study [46]. However, this meta-analysis reports conflicting observations with reference to corneal nerve features between different studies. Though corneal nerve feature-related changes such as increased tortuosity, beading, looping, and decreased nerve fibre density, etc., have been reported in various studies using IVCM [22,43], we did not find a significant difference across these nerve parameters assessed in the SBNP across the groups of our cohort. Possible ethnicity variation and measurement strategies (manual versus algorithm based) could contribute to differences in observations. Microneuroma-like structures, which are terminal enlargements of nerve endings with variable shape and hyperreflectivity [47], can be observed in the sub-basal nerve plexus or stroma of the cornea [35,43]. We found a significantly higher frequency of microneuroma-like structures among the subjects in the highly symptomatic D^++^S^+/-^ (group 4) compared to the other groups in our study. In subjects with the classic presentation of “pain without stain” consistent with NCP, the IVCM analysis revealed a higher proportion of microneuroma-like structures, similar to previous reports [34,35]. Our cohort also had a few asymptomatic subjects who were found to have microneuroma-like structures in their sub-basal nerve plexus, contrary to previous reports that did not show this feature in normal subjects [35]. This suggests that even though the microneuroma-like structures are strongly associated with NCP, the patient symptoms may be dependent on an interplay between inflammatory, structural, and neuronal components.

In addition to their role in modulating inflammation on the ocular surface, the dysregulation of certain molecular factors can have nociceptive potential as well. We have previously demonstrated that an altered balance between the pro- and anti-nociceptive tear soluble factors can contribute to the increased symptomatology in DED [36]. It has been shown that the ocular neurosensory pathway has various structural and molecular components and dysregulation of any of these can result in corneal hyperalgesia [48]. Inflammatory factors can sensitize thermoreceptors and mechano-nociceptors and reduce the threshold to pain stimuli [49]. Receptors of IL-17A, have been shown to be expressed by nociceptor neurons and, hence, play a role in pain perception by the altered expression of TRPV4 channels [50]. We found IL-17A, a pro-nociceptive factor, significantly increased in the D^+^S^+^ and D^++^S^+/-^ groups, with much higher levels in the latter. VEGF-A, in addition to being an angiogenic factor, also has anti-nociceptive potential [51,52]. In our study we found that the levels of anti-nociceptive factor VEGF-A were reduced in the D^+^S^+^ group, suggesting a tip in balance towards increased pro-nociception in these groups. The increased symptomatology in these groups could be due to this altered balance between pro- and anti-nociceptive factors on the ocular surface. It is pertinent that multicentric studies across different ethnicities and larger cohort be conducted to validate our findings to provide a clinically actionable algorithm that uses these patient specific corneal features for stratifying patients with ocular surface pain. Treatment targeted at improving the nociceptive balance could help improve patient symptoms and long-term comfort [34].

Currently, the treatment options for patients presenting with discordant signs versus symptoms are limited to the following: (a) artificial tear supplements along with anti-inflammatory agents such as topical steroids, (b) nonsteroidal medications such as cyclosporine A and tacrolimus, and (c) biologicals such as Anakinra (human IL-1 receptor antagonist). Further, autologous serum eye drops have also been shown to have a positive effect in some cases as they contain multiple factors such as nerve growth factor (NGF) and epidermal growth factor (EGF) that support nerve growth and epithelial health. In patients who have a central component of pain, systemic medications such as tricyclic antidepressants (e.g., amitriptyline, nortriptyline), anticonvulsants (e.g., Gabapentin, carbamazepine), and serotonin uptake inhibitors (e.g., Duloxetine and venlafaxine) may help relieve symptoms [34].

## 5. Conclusions

**Figure 12:**
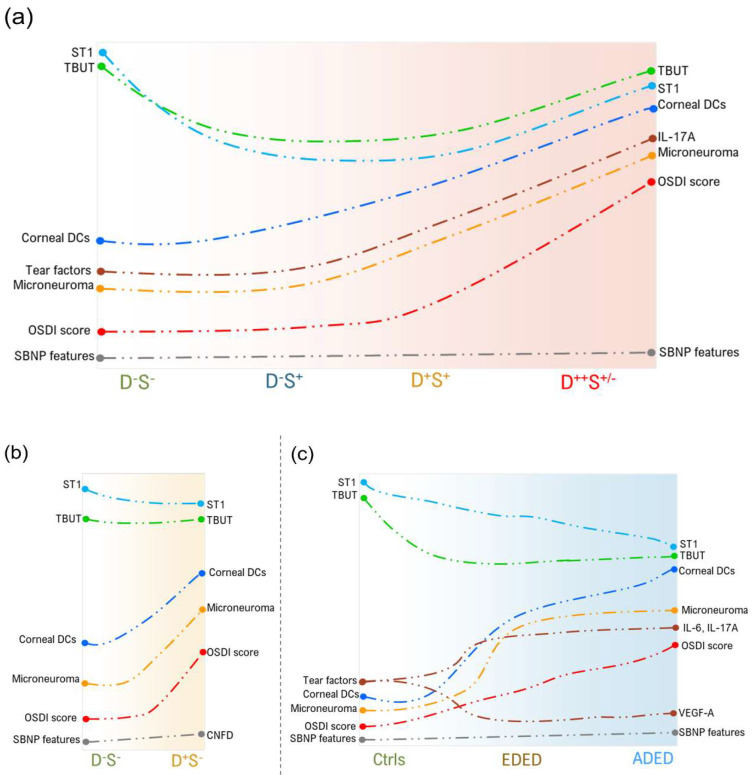
Schematic representation of signs, symptoms, confocal microscopy features, and tear fluid factors in in subjects categorized based on discomfort concordance to signs (DCS), grouping strategy, and dry eye disease types. (**a**) Line graph representation indicate the patterns (based on statistically significant observations) in ocular surface disease index (OSDI) score, tear break up time (TBUT), Schirmer’s test (ST1), corneal dendritic cell (DCs) density, sub-basal nerve plex (SBNP) features, microneuroma-like structures (microneuroma), and tear fluid factors across the different categories based on DCS grouping strategy as described in Figure 1. (**b**) Line graph representation indicate the patterns (based on statistically significant observations) in ocular surface disease index (OSDI) score, tear break up time (TBUT), Schirmer’s test (ST1), corneal dendritic cell (DCs) density, sub-basal nerve plex (SBNP) features, microneuroma-like structures (microneuroma), and tear fluid factors in subjects with discomfort but no signs. D^-^S^-^ indicates subjects without discomfort/symptoms and signs and D^+^S^-^ indicates subjects with discomfort/symptoms but no signs. (**c**) Line graph representation indicates the patterns (based on statistically significant observations) in ocular surface disease index (OSDI) score, tear break up time (TBUT), Schirmer’s test (ST1), corneal dendritic cell (DCs) density, sub-basal nerve plex (SBNP) features, microneuroma-like structures (microneuroma), and tear fluid factors in controls and in subjects with evaporative dry eye disease (EDED) or aqueous deficient dry eye disease (ADED). Controls are subjects without discomfort/symptoms and signs (D^-^S^-^).

The assessment of ocular pain based on questionnaires and correlating it to clinical metrics and confocal features such as cDC density and microneuroma-like structures can help clinicians better classify the condition and aid in customized treatment planning. The discordance between patient reported ocular surface pain/discomfort and clinical signs is associated with enhanced cDC, presence of microneuroma-like structures imaged by IVCM, as well as increased IL-17A (Figure 12). This study highlights the importance of scoring the disparity in symptoms and signs and IVCM as important clinical tools to stratify patients for targeted treatment.

## Figures and Tables

**Table 1:** Tear fluid secreted factors levels in subjects with discomfort but no signs.

	D^-^S^-^			D^+^S^-^			*p* Value
	Mean	SD	SEM	Mean	SD	SEM	
IL-1⍺	44	45	8	38	41	15	0.601
IL-1β	5.9	11.5	2.2	7.3	11.9	4.2	0.522
IL-6	0.0	0.0	0.0	0.0	0.0	0.0	1.000
IL-8	272	281	53	247	178	63	0.950
IL-10	0.2	0.3	0.1	0.3	0.5	0.2	0.830
IL-17A	1.2	2.3	0.4	64.3	176.5	62.4	0.250
IL-17F	74	236	45	76	195	69	0.929
TNF-⍺	0.5	0.9	0.2	0.7	1.1	0.4	0.865
IFN-γ	2.7	6.1	1.1	117.3	324.7	114.8	0.504
RANTES	102	131	25	48	58	21	0.196
MCP-1	300	290	55	320	202	72	0.672
VEGF-A	1275	822	155	794	829	293	0.075
ICAM-1	2793	2710	512	1449	759	268	0.305

D^-^S^-^ (subjects without discomfort/symptoms and without signs—OSDI score: <12, TBUT ≥ 10 s, Schirmer’s test 1: ≥10 mm/5 min); D^+^S^-^ (subjects with discomfort/symptoms and without signs—OSDI score: >12, TBUT ≥ 10 s, Schirmer’s test 1: ≥10 mm/5 min); SD—standard deviation; SEM—standard error mean; D^-^S^-^ (n = 28 eyes); D^+^S^-^ (n = 8 eyes); *p* < 0.05 is statistically significant, Mann–Whitney Test.

**Table 2:** Tear fluid secreted factors levels in healthy subjects and in patients with evaporative or aqueous deficient dry eye disease.

Analytes (pg/mL)	Controls			EDED			ADED			*p* Value		
	Mean	SD	SEM	Mean	SD	SEM	Mean	SD	SEM	Ctrls vs. EDED	Ctrls vs. ADED	EDED vs. ADED
IL-1⍺	44	45	8	33	42	6	29	13	5	0.095	0.789	0.474
IL-1β	6	12	2	4	6	1	7	8	3	0.254	0.208	0.421
IL-6	0	0	0	6	22	3	42	68	28	0.022	<0.0001	0.499
IL-8	272	281	53	287	199	30	632	806	329	0.395	0.092	0.209
IL-10	0	0	0	0	0	0	0	0	0	0.217	0.997	0.507
IL-17A	1	2	0	9	17	2	3	2	1	0.003	<0.0001	0.583
IL-17F	74	236	45	62	195	29	0	0	0	0.713	0.440	0.583
TNF-⍺	0	1	0	1	1	0	1	1	0	0.480	0.441	0.447
IFN-γ	3	6	1	9	21	3	0	0	0	0.427	0.228	0.113
RANTES	102	131	25	188	483	72	381	242	99	0.812	0.002	0.013
MCP-1	300	290	55	166	232	35	261	340	139	0.008	0.388	0.704
VEGF-A	1275	822	155	971	1303	194	446	326	133	0.009	0.008	0.663
ICAM-1	2793	2710	512	2526	4162	620	2610	1238	505	0.220	0.713	0.254

Controls: n = 28 eyes; EDED (Evaporative dry eye disease): n = 45 eyes; ADED (Aqueous deficient dry eye disease): n = 6 eyes; SD—standard deviation; SEM—standard error mean; *p* < 0.05 is statistically significant, Mann–Whitney Test.

## Data Availability

Data is available upon request.

## Supplementary Materials

The following supporting information can be downloaded at: https://www.mdpi.com/article/10.3390/jcm11092407/s1, Figure S1: Sub-basal nerve plexus features profile in subjects with dry eye disease; Figure S2: Frequency of microneuroma-like structures in subjects with dry eye disease.

## References

[1] Mehra D., Cohen N.K., Galor A. (2020). Ocular Surface Pain: A Narrative Review. Ophthalmol. Ther..

[2] Dana R., Meunier J., Markowitz J.T., Joseph C., Siffel C. (2020). Patient-Reported Burden of Dry Eye Disease in the United States: Results of an Online Cross-Sectional Survey. Am. J. Ophthalmol..

[3] Dermer H., Lent-Schochet D., Theotoka D., Paba C., Cheema A.A., Kim R.S., Galor A. (2020). A Review of Management Strategies for Nociceptive and Neuropathic Ocular Surface Pain. Drugs.

[4] Morone N.E., Weiner D.K. (2013). Pain as the fifth vital sign: Exposing the vital need for pain education. Clin. Ther..

[5] Shaheen B.S., Bakir M., Jain S. (2014). Corneal nerves in health and disease. Surv. Ophthalmol..

[6] Jensen T.S., Baron R., Haanpää M., Kalso E., Loeser J.D., Rice A.S., Treede R.-D. (2011). A new definition of neuropathic pain. Pain.

[7] Galor A. (2019). Painful Dry Eye Symptoms: A Nerve Problem or a Tear Problem?. Ophthalmology.

[8] Jacobs D.S. (2017). Diagnosis and Treatment of Ocular Pain: The Ophthalmologist’s Perspective. Curr. Ophthalmol. Rep..

[9] Galor A., Feuer W.J., Lee D.J., Florez H., Venincasa V., Perez V.L. (2013). Ocular surface parameters in older male veterans. Investig. Opthalmol. Vis. Sci..

[10] Stapleton F., Alves M., Bunya V.Y., Jalbert I., Lekhanont K., Malet F., Na K.-S., Schaumberg D., Uchino M., Vehof J. (2017). TFOS DEWS II Epidemiology Report. Ocul. Surf..

[11] Vehof J., Kozareva D., Hysi P.G., Harris J., Nessa A., Williams F.K., Bennett D.L.H., McMahon S.B., Fahy S.J., Direk K. (2013). Relationship between dry eye symptoms and pain sensitivity. JAMA Ophthalmol..

[12] Rosenthal P., Baran I., Jacobs D.S. (2009). Corneal pain without stain: Is it real?. Ocul. Surf..

[13] Galor A., Levitt R.C., Felix E.R., Martin E.R., Sarantopoulos C.D. (2015). Neuropathic ocular pain: An important yet underevaluated feature of dry eye. Eye.

[14] Galor A., Moein H.-R., Lee C., Rodriguez A., Felix E., Sarantopoulos K.D., Levitt R.C. (2018). Neuropathic pain and dry eye. Ocul. Surf..

[15] Nichols K.K., Nichols J.J., Mph M., Mitchell G.L. (2004). The lack of association between signs and symptoms in patients with dry eye disease. Cornea.

[16] Ong E.S., Felix E., Levitt R.C., Feuer W.J., Sarantopoulos C.D., Galor A. (2018). Epidemiology of discordance between symptoms and signs of dry eye. Br. J. Ophthalmol..

[17] Bron A.J., de Paiva C.S., Chauhan S.K., Bonini S., Gabison E.E., Jain S., Knop E., Markoulli M., Ogawa Y., Perez V. (2017). TFOS DEWS II pathophysiology report. Ocul. Surf..

[18] Belmonte C., Acosta M.C., Merayo-Lloves J., Gallar J. (2015). What Causes Eye Pain?. Curr. Ophthalmol. Rep..

[19] He J., Ogawa Y., Mukai S., Saijo-Ban Y., Kamoi M., Uchino M., Yamane M., Ozawa N., Fukui M., Mori T. (2017). In Vivo Confocal Microscopy Evaluation of Ocular Surface with Graft-Versus-Host Disease-Related Dry Eye Disease. Sci. Rep..

[20] Kheirkhah A., Darabad R.R., Cruzat A., Hajrasouliha A.R., Witkin D., Wong N., Dana R., Hamrah P. (2015). Corneal Epithelial Immune Dendritic Cell Alterations in Subtypes of Dry Eye Disease: A Pilot In Vivo Confocal Microscopic Study. Investig. Opthalmol. Vis. Sci..

[21] Giannaccare G., Pellegrini M., Sebastiani S., Moscardelli F., Versura P., Campos E.C. (2019). In vivo confocal microscopy morphometric analysis of corneal subbasal nerve plexus in dry eye disease using newly developed fully automated system. Graefe’s Arch. Clin. Exp. Ophthalmol..

[22] Tervo T.M., Moilanen J.A.O., Rosenberg M.E., Tuominen I.S.J., Valle T., Vesaluoma M.H. (2002). In vivo confocal microscopy for studying corneal diseases and conditions associated with corneal nerve damage. Adv. Exp. Med. Biol..

[23] Wolffsohn J.S., Arita R., Chalmers R., Djalilian A., Dogru M., Dumbleton K., Gupta P.K., Karpecki P., Lazreg S., Pult H. (2017). TFOS DEWS II Diagnostic Methodology report. Ocul. Surf..

[24] Satitpitakul V., Kheirkhah A., Crnej A., Hamrah P., Dana R. (2017). Determinants of Ocular Pain Severity in Patients with Dry Eye Disease. Am. J. Ophthalmol..

[25] Miller K.L., Walt J.G., Mink D.R., Satram-Hoang S., Wilson S.E., Perry H.D., Asbell P.A., Pflugfelder S.C. (2010). Minimal clinically important difference for the ocular surface disease index. Arch. Ophthalmol..

[26] Craig J.P., Nichols K.K., Akpek E.K., Caffery B., Dua H.S., Joo C.-K., Liu Z., Nelson J.D., Nichols J.J., Tsubota K. (2017). TFOS DEWS II Definition and Classification Report. Ocul. Surf..

[27] Lemp M.A. (2007). The definition and classification of dry eye disease: Report of the Definition and Classification Subcommittee of the International Dry Eye WorkShop (2007). Ocul. Surf..

[28] Mantopoulos D., Cruzat A., Hamrah P. (2010). In vivo imaging of corneal inflammation: New tools for clinical practice and research. Semin. Ophthalmol..

[29] Patel D., McGhee C.N. (2008). In vivo confocal microscopy of human corneal nerves in health, in ocular and systemic disease, and following corneal surgery: A review. Br. J. Ophthalmol..

[30] Lagali N.S., Badian R.A., Liu X., Feldreich T.R., Arnlov J., Utheim T.P., Dahlin L.B., Rolandsson O. (2018). Dendritic cell maturation in the corneal epithelium with onset of type 2 diabetes is associated with tumor necrosis factor receptor superfamily member 9. Sci. Rep..

[31] Shetty R., Deshmukh R., Shroff R., Dedhiya C., Jayadev C. (2018). Subbasal Nerve Plexus Changes in Chronic Migraine. Cornea.

[32] Shetty R., Sethu S., Deshmukh R., Deshpande K., Ghosh A., Agrawal A., Shroff R. (2016). Corneal Dendritic Cell Density Is Associated with Subbasal Nerve Plexus Features, Ocular Surface Disease Index, and Serum Vitamin D in Evaporative Dry Eye Disease. BioMed Res. Int..

[33] Dieckmann G., Goyal S., Hamrah P. (2017). Neuropathic Corneal Pain: Approaches for Management. Ophthalmology.

[34] Goyal S., Hamrah P. (2016). Understanding Neuropathic Corneal Pain—Gaps and Current Therapeutic Approaches. Semin. Ophthalmol..

[35] Ross A.R., Al-Aqaba M.A., Almaazmi A., Messina M., Nubile M., Mastropasqua L., Dua H.S., Said D.G. (2020). Clinical and in vivo confocal microscopic features of neuropathic corneal pain. Br. J. Ophthalmol..

[36] Khamar P., Nair A.P., Shetty R., Vaidya T., Subramani M., Ponnalagu M., Dhamodaran K., D’Souza S., Ghosh A., Pahuja N. (2019). Dysregulated Tear Fluid Nociception-Associated Factors, Corneal Dendritic Cell Density, and Vitamin D Levels in Evaporative Dry Eye. Investig. Ophthalmol. Vis. Sci..

[37] Hucho T., Levine J.D. (2007). Signaling pathways in sensitization: Toward a nociceptor cell biology. Neuron.

[38] Chiu I.M., von Hehn C.A., Woolf C.J. (2012). Neurogenic inflammation and the peripheral nervous system in host defense and immunopathology. Nat. Neurosci..

[39] Dana M.R., Hamrah P. (2002). Role of immunity and inflammation in corneal and ocular surface disease associated with dry eye. Adv. Exp. Med. Biol..

[40] Muller L.J., Marfurt C.F., Kruse F., Tervo T.M. (2003). Corneal nerves: Structure, contents and function. Exp. Eye Res..

[41] McKay T.B., Seyed-Razavi Y., Ghezzi C.E., Dieckmann G., Nieland T.J.F., Cairns D.M., Pollard R.E., Hamrah P., Kaplan D.L. (2019). Corneal pain and experimental model development. Prog. Retin. Eye Res..

[42] Austin P.J., Moalem-Taylor G. (2010). The neuro-immune balance in neuropathic pain: Involvement of inflammatory immune cells, immune-like glial cells and cytokines. J. Neuroimmunol..

[43] Aggarwal S., Kheirkhah A., Cavalcanti B.M., Cruzat A., Colon C., Brown E., Borsook D., Pruss H., Hamrah P. (2015). Autologous Serum Tears for Treatment of Photoallodynia in Patients with Corneal Neuropathy: Efficacy and Evaluation with In Vivo Confocal Microscopy. Ocul. Surf..

[44] Hattori T., Takahashi H., Dana R. (2016). Novel Insights Into the Immunoregulatory Function and Localization of Dendritic Cells. Cornea.

[45] Dastjerdi M.H., Dana R. (2009). Corneal nerve alterations in dry eye-associated ocular surface disease. Int. Ophthalmol. Clin..

[46] Xu J., Chen P., Yu C., Liu Y., Hu S., Di G. (2021). In vivo Confocal Microscopic Evaluation of Corneal Dendritic Cell Density and Subbasal Nerve Parameters in Dry Eye Patients: A Systematic Review and Meta-analysis. Front. Med..

[47] Moein H.R., Akhlaq A., Dieckmann G., Abbouda A., Pondelis N., Salem Z., Muller R.T., Cruzat A., Cavalcanti B.M., Jamali A. (2020). Visualization of microneuromas by using in vivo confocal microscopy: An objective biomarker for the diagnosis of neuropathic corneal pain?. Ocul. Surf..

[48] Belmonte C., Nichols J.J., Cox S.M., Brock J.A., Begley C.G., Bereiter D.A., Dartt D.A., Galor A., Hamrah P., Ivanusic J.J. (2017). TFOS DEWS II pain and sensation report. Ocul. Surf..

[49] Pinho-Ribeiro F.A., Verri W.A., Chiu I.M. (2017). Nociceptor Sensory Neuron-Immune Interactions in Pain and Inflammation. Trends Immunol..

[50] Kim C.F., Moalem-Taylor G. (2011). Interleukin-17 contributes to neuroinflammation and neuropathic pain following peripheral nerve injury in mice. J. Pain.

[51] Hulse R.P., Beazley-Long N., Ved N., Bestall S.M., Riaz H., Singhal P., Ballmer Hofer K., Harper S.J., Bates D.O., Donaldson L.F. (2015). Vascular endothelial growth factor-A165b prevents diabetic neuropathic pain and sensory neuronal degeneration. Clin. Sci..

[52] Hulse R.P. (2017). Role of VEGF-A in chronic pain. Oncotarget.

