# Supplementary material for: Corneal Confocal Microscopy Features and Tear Molecular Profile in Study Participants with Discordance between Ocular Surface Disease Clinical Signs and Discomfort
Source: J Clin Med. 2022 Apr 25;11(9):2407. doi: 10.3390/jcm11092407 (PMC9099769; doi:10.3390/jcm11092407)
Supplement: Supplementary file 1 [file jcm-11-02407-s001.zip › jcm-1642131-supplementary.pdf]

Supplementary Figure S1

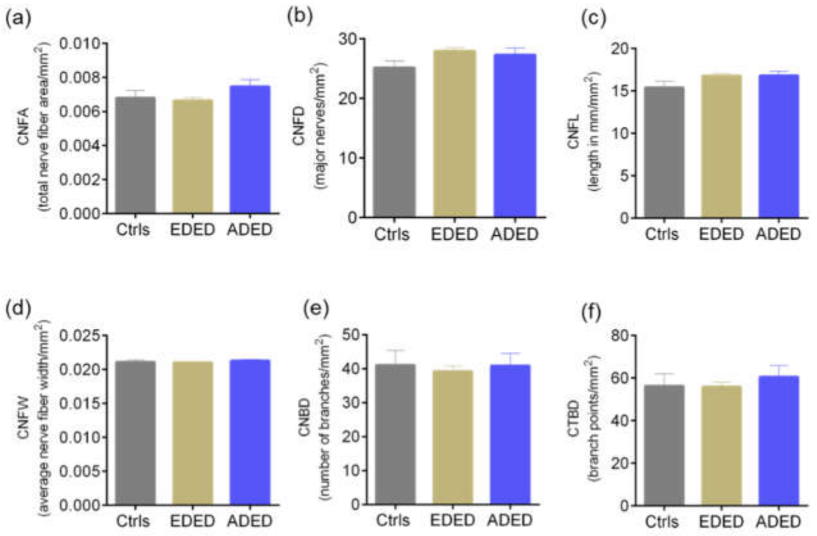

Figure S1. Sub-basal nerve plexus features profile in subjects with dry eye disease. Bar graphs represent (**a**) corneal nerve fibre area—CNFA, (**b**) corneal nerve fibre density—CNFD, (**c**) corneal nerve fibre length—CNFL, (**d**) corneal nerve fibre width—CNFW, (**e**) corneal nerve branch density—CNBD and (**f**) corneal total branch density—CTBD determined using laser scanning in vivo confocal microscopic images in controls and in subjects with evaporative dry eye disease (EDED) or aqueous deficient dry eye disease (ADED). Controls are subjects without discomfort/symptoms and signs (D-S-). The number of eyes analysed for corneal dendritic cell density are as follows—Controls (Ctrls): n = 52 eyes; EDED: n = 144 eyes; ADED: n = 48 eyes. The number of eyes analysed for sub-basal nerve plexus features are as follows—Controls (Ctrls): n = 29 eyes; EDED: n = 114 eyes; ADED: n = 32 eyes. Kruskal-Wallis test with Dunn's multiple comparisons test was performed.

Supplementary Figure S2

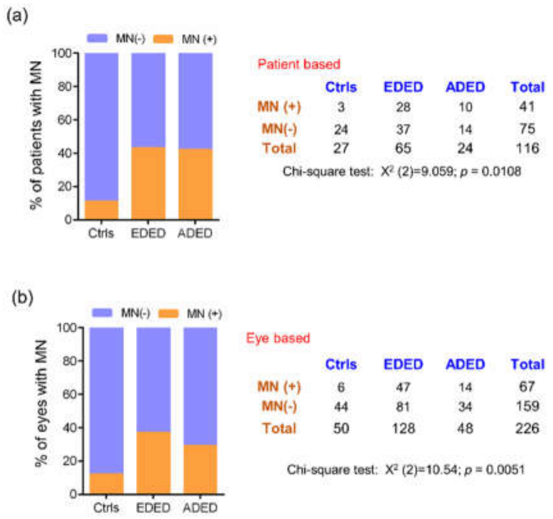

Figure S2. Frequency of microneuroma-like structures in subjects with dry eye disease. Stacked bar graphs represent (**a**) percentage of patients with and without microneuroma-like structures determined in laser scanning in vivo confocal microscopy images in controls and in subjects with evaporative dry eye disease (EDED) or aqueous deficient dry eye disease (ADED). Controls are subjects without discomfort/symptoms and signs (D^-S^-). The adjacent table provides the absolute number of patients with and without microneuroma-like structures in the different groups. (**b**) percentage of eyes with and without microneuroma-like structures determined in laser scanning in vivo confocal microscopy images in controls and in subjects with evaporative dry eye disease (EDED) or aqueous deficient dry eye disease (ADED). Controls are subjects without discomfort/symptoms and signs (D^-S^-). The adjacent table provides the absolute number of eyes with and without microneuroma-like structures in the different groups. Chi-square test was performed to determine the statistical significance of the difference in the frequency of microneuroma-like structures between the groups. $p < 0.05$ is considered to be statistically significant. MN—microneuroma-like structures.
